# Efficacy of Probiotics in the Management of Irritable Bowel Syndrome: A Systematic Review and Meta-Analysis

**DOI:** 10.7759/cureus.75954

**Published:** 2024-12-18

**Authors:** Bandar A Almabruk, Ali A Bajafar, Ahmed N Mohamed, Saleh A Al-Zahrani, Naif M Albishi, Rafeef Aljarwan, Rola A Aljaser, Lama I Alghamdi, Turki S Almutairi, Almas S Alsolami, Joud K Alghamdi

**Affiliations:** 1 Internal Medicine, King Salman Hospital, Riyadh, SAU; 2 General Practice, King Abdullah Medical Complex, Jeddah, SAU; 3 Critical Care Medicine, SEHA Salma Rehabilitation Hospital, Abu Dhabi, ARE; 4 College of Medicine, King Saud Bin Abdulaziz University for Health Sciences, Jeddah, SAU; 5 Nursing, Imam Abdulrahman Bin Faisal University, Dammam, SAU; 6 College of Medicine, King Saud Bin Abdulaziz University for Health Sciences, Riyadh, SAU; 7 Medicine, Batterjee Medical College, Jeddah, SAU; 8 College of Medicine and Surgery, Taif University, Taif, SAU; 9 College of Applied Medical Sciences, King Saud Bin Abdulaziz University for Health Sciences, Jeddah, SAU; 10 Faculty of Medicine, King Abdulaziz University Hospital, Jeddah, SAU

**Keywords:** abdominal pain, gastrointestinal microbiome, irritable bowel syndrome, probiotics, quality of life

## Abstract

Irritable bowel syndrome (IBS) significantly impacts quality of life. Probiotics offer relief by modulating gut microbiota, but variability in outcomes necessitates a systematic evaluation of their efficacy. This study aims to evaluate the efficacy of probiotics in improving symptoms of IBS through a systematic review and meta-analysis. A comprehensive search of PubMed and Google Scholar identified studies published between 2014 and 2018. Inclusion criteria focused on randomized controlled trials evaluating probiotics in adult IBS patients diagnosed using standardized criteria. Statistical analysis utilized random effects models to account for heterogeneity, with subgroup analysis performed for IBS subtypes. This review included 23 studies involving 3,288 participants. Probiotics significantly reduced abdominal pain (mean difference = -1.66, 95% CI = -2.39 to -0.93, p < 0.0001) and bloating (mean difference = -2.13, 95% CI = -3.96 to -0.30, p = 0.0224). Improvement in stool habits was significant (mean difference = -1.52, 95% CI = -2.15 to -0.88, p < 0.0001), particularly in diarrhea-predominant IBS. Quality of life improved significantly, with a mean increase of 8.77 points (95% CI = 0.91 to 16.64, p = 0.028). Adverse events were mild and infrequent. However, heterogeneity was high (I² > 90%), reflecting variability in study protocols. Probiotics are effective in reducing IBS symptoms and improving quality of life, mainly in diarrhea-predominant IBS. More research should be conducted that focuses on standardized, long-term trials to refine treatment strategies.

## Introduction and background

Irritable bowel syndrome (IBS) is a common functional gastrointestinal disorder characterized by recurrent abdominal pain and altered bowel habits, which can manifest as diarrhea, constipation, or a mix of both [1]. IBS is not life-threatening but significantly affects the quality of life, productivity, and mental well-being of those affected [2]. The global pooled prevalence of IBS is 15.0%, with significant variation between regions. The highest prevalence is 18.9% in South America, while the lowest is 11.0% in Southeast Asia, indicating that rates can exceed 20% in certain areas [3].

The role of gut microbiota in IBS has gained increasing attention in recent years. Studies have demonstrated that individuals with IBS often exhibit gut dysbiosis [4,5]. This imbalance (dysbiosis) is believed to contribute to symptoms such as bloating, abdominal pain, and irregular bowel movements. As a result, researchers and clinicians have explored therapies aimed at restoring the gut microbiota, with probiotics emerging as a promising intervention [6]. Probiotics are thought to improve gut health by enhancing microbial diversity and modulating inflammatory responses [7].

Recent studies and clinical trials have been conducted to evaluate the efficacy of probiotics in managing IBS symptoms. Specific strains such as *Lactobacillus rhamnosus*, *Bifidobacterium infantis*, and *Saccharomyces boulardii* have been studied extensively, but their individual effects on IBS symptoms remain inconclusive [8-11].

Despite the growing body of literature on probiotics for IBS, several gaps and limitations exist. One important limitation is the heterogeneity of existing studies, including variations in diagnostic criteria, participant demographics, and treatment protocols. Many studies have small sample sizes and lack long-term follow-up, making it difficult to generalize findings. These limitations show the need for systematic reviews and meta-analysis to synthesize evidence and provide a clearer understanding of the role of probiotics in IBS management. This study aims to address these gaps by systematically reviewing and analyzing published research on probiotics for IBS. By pooling data from multiple studies, we can assess the overall efficacy of probiotics and identify which strains or combinations are most effective. This analysis will explore factors influencing treatment outcomes, such as patient subgroups, duration of therapy, and dosage levels.

## Review

Material and methods

This systematic review and meta-analysis adhered to the Preferred Reporting Items for Systematic Reviews and Meta-Analyses (PRISMA) guidelines to ensure transparency and methodological rigor in study identification, selection, and analysis.

Literature Search Strategy

A comprehensive literature search was conducted across two major electronic databases, including PubMed and Google Scholar, to identify relevant studies published between 2014 and 2018. The search strategy combined the use of Boolean operators ("AND" and "OR") with specific keywords to maximize the retrieval of relevant literature. The search terms included “irritable bowel syndrome”, “IBS”, “probiotics”, “efficacy”, “gut microbiota”, and “randomized controlled trials”. For example, the following query was employed: ("Irritable Bowel Syndrome" OR "IBS") AND ("Probiotics" OR "Gut Microbiota") AND ("Efficacy" OR "Treatment").

Inclusion and Exclusion Criteria

Studies were included if they involved adult participants diagnosed with IBS based on recognized diagnostic criteria, such as the Rome criteria. Only randomized controlled trials (RCTs) and clinical trials evaluating the efficacy of probiotics as a primary intervention were considered. Eligible studies needed to report measurable outcomes related to IBS symptom improvement, such as reductions in abdominal pain, bloating, stool consistency issues, or overall symptom severity scores. Only studies published in English and accessible in full text were included to ensure the quality and reliability of the data.

Studies were excluded if they focused on pediatric populations, as the pathophysiology and treatment response in children differs significantly from adults. Research evaluating interventions other than probiotics, or those combining multiple therapies without isolating the specific effects of probiotics, were also excluded. Observational studies, case reports, reviews, and meta-analyses without primary data were also excluded, as they did not provide original results suitable for pooled analysis. Articles with incomplete or ambiguous data that could not be clarified were excluded as well. These criteria were designed to minimize bias, enhance comparability, and ensure the synthesis of evidence regarding the efficacy of probiotics in IBS management.

Study Selection

**Figure 1:**
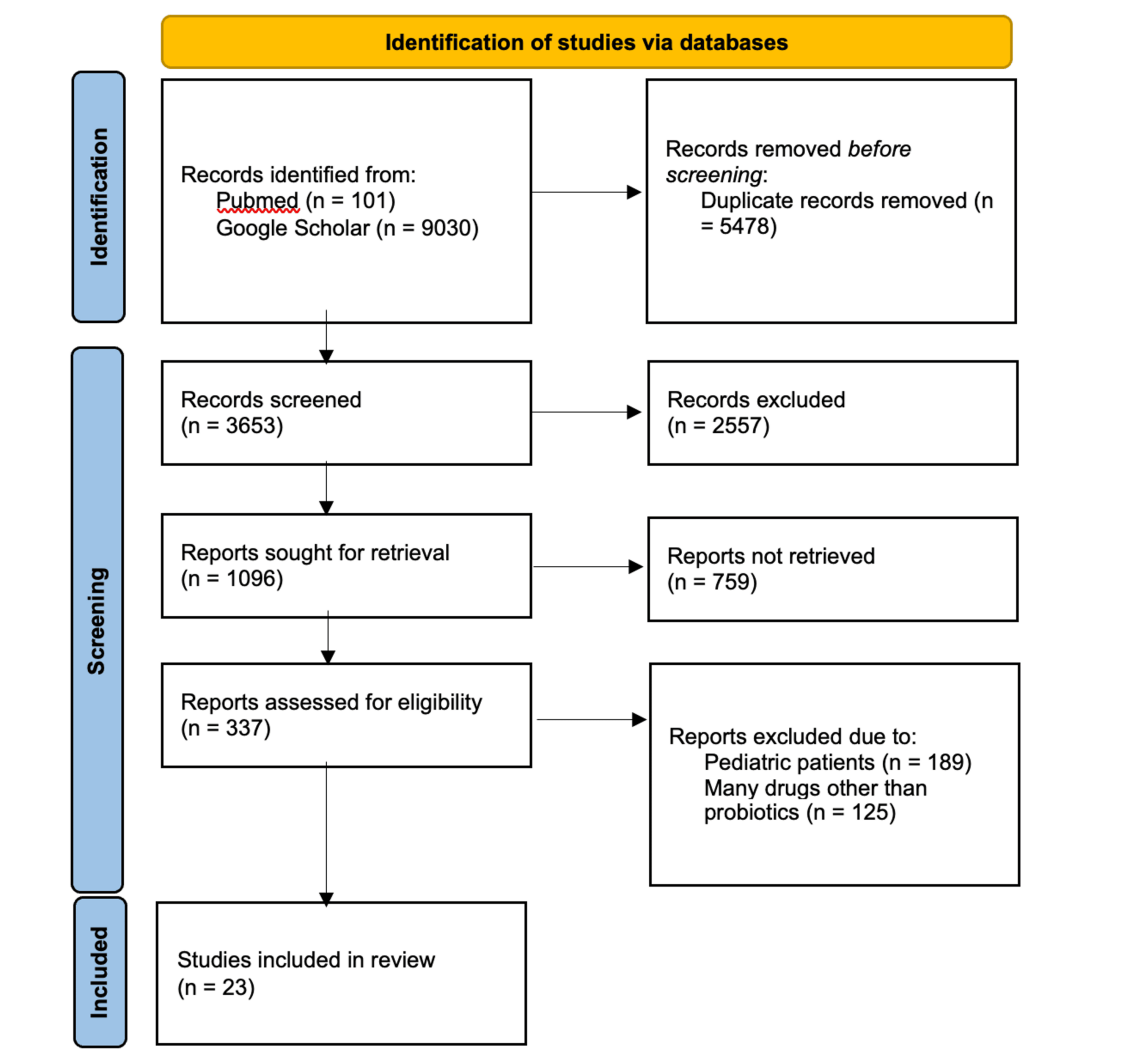
PRISMA flowchart showing the selection criteria and inclusion of studies. PRISMA: Preferred Reporting Items for Systematic Reviews and Meta-Analyses [12].

The selection process for studies is outlined in Figure 1. Initially, 9,131 records were identified through database searches, with 5,478 duplicates removed before screening. The titles and abstracts of 3,653 records were then assessed for relevance, resulting in the exclusion of 2,557 articles due to irrelevance or lack of focus on probiotics and IBS. Subsequently, 1,096 full-text articles were evaluated for eligibility. Of these, 759 articles were excluded, primarily because they included pediatric populations (n = 189) or evaluated multiple therapies without isolating the effects of probiotics (n = 125). Finally, 23 studies met the inclusion criteria and were included in the final analysis.

Data Extraction and Management

Two independent reviewers screened titles, abstracts, and full-text articles for eligibility. Discrepancies were resolved through discussion or consultation with a third reviewer. Data extraction was performed using a standardized form that captured the study's author, year, design, sample size, participant demographics, IBS subtypes, diagnostic criteria, probiotic strains and dosages, treatment duration, and reported outcomes. Studies meeting all inclusion criteria were included in the final analysis.

Quality Assessment of Included Studies

**Figure 2:**
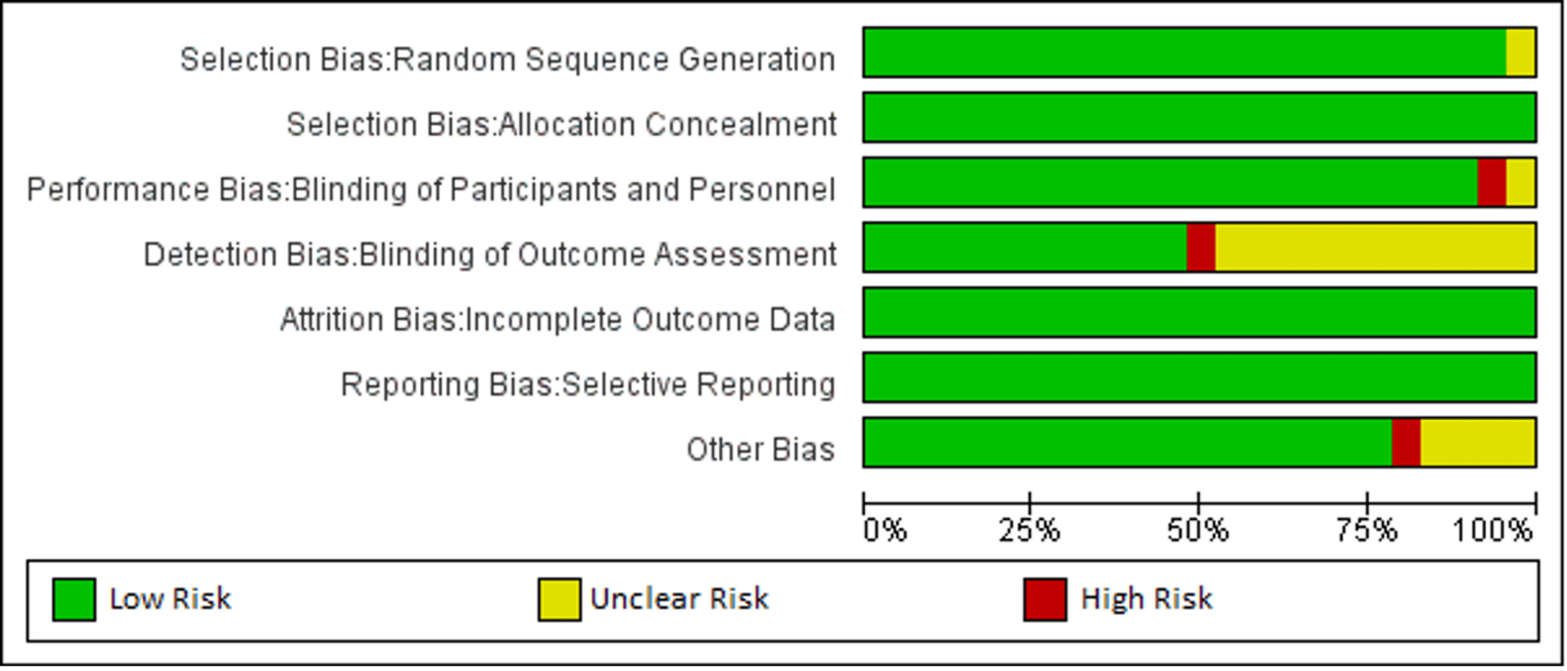
Risk of bias graph: review authors' judgments about each risk of bias item presented as percentages across all included studies.

**Figure 3:**
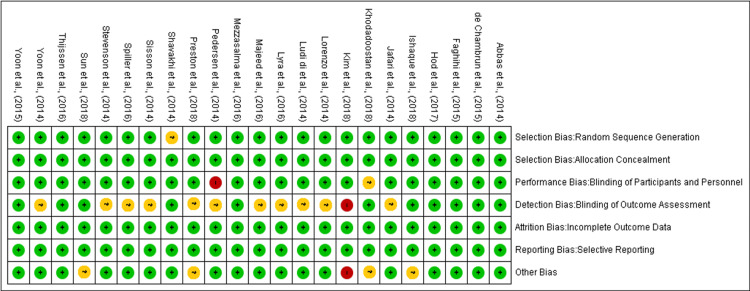
Risk of bias summary: review authors' judgments about each risk of bias item for each included study. References [13-35].

The methodological quality of our study was meticulously assessed using the Cochrane Risk of Bias Assessment Tool. This assessment was conducted by three independent reviewers, ensuring a robust evaluation process. In cases where discrepancies arose, consensus was reached among the reviewers through mutual discussion. If consensus could not be achieved, a third party was involved to facilitate conflict resolution, ensuring impartiality and accuracy in the evaluation process. Figure 2 demonstrates the distribution of low, unclear, and high risks across several methodological domains for the included studies. The majority achieved low-risk status for random sequence generation and allocation concealment, indicating well-implemented randomization and effective concealment of allocation sequences. Nevertheless, some studies were classified as having an unclear risk in random sequence generation, mainly due to inadequate reporting on the randomization process. Performance bias was generally low, suggesting effective blinding of participants and personnel across many studies, although a few exhibited high risk, likely from insufficient blinding procedures, which could affect the equality of treatment across intervention groups. Detection bias varied, with a mixture of low and unclear risks due to inconsistencies in blinding outcome assessors, particularly for subjective outcomes like symptom severity or quality of life, which could skew results. Most studies managed attrition bias well, indicating sound handling of incomplete data and employing intention-to-treat analyses, but a few had unclear risks due to poorly reported dropout rates and their impacts. Reporting bias was mostly low, reflecting thorough outcome reporting, yet selective reporting issues were hinted at in a minority. The "other biases" category also showed mainly low risk, affirming adherence to stringent study protocols, although occasional high risks pointed to potential unmeasured confounders or methodological shortcomings. Figure 3 shows that the risk of bias summary chart visualizes the assessment of several RCTs on various bias criteria according to the Cochrane Collaboration tool. Studies by Abbas et al. (2014) [13] and de Chambrun et al. (2015) [14] exhibit low risk across all domains, indicating robust methodologies. Conversely, studies by Khodadoostan et al. (2018) [15] and Kim et al. (2018) [16] show a mix of unclear and high-risk judgments, suggesting potential biases in areas like blinding and allocation concealment.

Results

This review included 23 studies [13-35], with a total sample size of 3,288 participants. Studies predominantly utilized an RCT design, ensuring high methodological quality. Recruitment was primarily conducted in secondary and tertiary care settings. Participant ages ranged from 31.9 to 59.3 years, representing a wide demographic range of adults affected by IBS. Gender distribution showed a higher prevalence of female participants, aligning with the known epidemiology of IBS; for instance, one study reported 292 females out of 391 participants. These studies collectively represent a dataset of IBS populations, ensuring a strong basis for evaluating the efficacy of probiotics (Table 1).

**Table 1:** Characteristics of included studies. NA: not applicable.

Author (year)	Design	Recruitment	Sample size	Age (years)	No. of males	No. of females
Abbas et al. (2014) [13]	Randomized controlled trial	Tertiary care	72	35.4 ± 11.9	53	19
Jafari et al. (2014) [17]	Randomized controlled trial	Secondary care	108	36.7 ± 11.5	43	65
Lorenzo et al. (2014) [18]	Randomized controlled trial	Tertiary care	84	46.8 ± 12.5	31	53
Ludidi et al. (2014) [19]	Randomized controlled trial	Secondary care	40	40.5 ± 14.4	13	27
Pedersen et al. (2014) [20]	Randomized controlled trial	Tertiary care	81	NA	25	56
Shavakhi et al. (2014) [21]	Randomized placebo-controlled trial	Tertiary care	129	36.2 ± 9.2	44	86
Sisson et al. (2014) [22]	Randomized controlled trial	Primary care and secondary care	186	38.3 ± 10.6	57	129
Stevenson et al. (2014) [23]	Randomized controlled trial	Secondary care	81	47.9 ± 13	2	79
Yoon et al. (2014) [24]	Randomized controlled trial	Tertiary care	49	44.5 ± 14.3	17	32
Faghihi et al. (2015) [25]	Randomized controlled trial	Secondary care	139	38 ± 13.3	NA	NA
de Chambrun et al. (2015) [14]	Randomized controlled trial	NA	179	44 ± 13.3	25	154
Yoon et al. (2015) [26]	Randomized placebo-controlled trial	Tertiary care	80	59.3 ± 12.2	43	37
Lyra et al. (2016) [27]	Clinical trial	Primary care	391	47.9 ± 12.9	99	292
Majeed et al. (2016) [28]	Randomized controlled trial	Tertiary care	36	35.8 ± 10.8	17	19
Mezzasalma et al. (2016) [29]	Randomized controlled trial	NA	150	37.4 ± 12.5	NA	NA
Spiller et al. (2016) [30]	Randomized placebo-controlled trial	Primary care and secondary care	379	45.3 ± 14.9	62	317
Thijssen et al. (2016) [31]	Randomized controlled trial	Secondary care and tertiary care	80	41.8 ± 14.1	25	55
Hod et al. (2017) [32]	Randomized controlled trial	Secondary and tertiary care	107	NA	0	107
Ishaque et al. (2018) [33]	Randomized controlled trial	Tertiary care	360	31.9 ± 9.9	281	79
Khodadoostan et al. (2018) [15]	Clinical trial	Secondary care and tertiary care	67	34.1 ± 11.0	43	24
Kim et al. (2018) [16]	Randomized placebo-controlled trial	Tertiary care	42	32.7 ± 6.6	25	17
Preston et al. (2018) [34]	Randomized controlled trial	Tertiary care	113	40.4 ± 13.5	68	45
Sun et al. (2018) [35]	Randomized controlled trial	Tertiary care	200	43.9 ± 12.7	116	84

Among the included studies, Rome III criteria were utilized in over 80% of cases, ensuring consistent diagnostic standards. Diarrhea-predominant IBS (IBS-D) was the most common subtype (reported in up to 100% of participants in several studies), followed by mixed and constipation-predominant IBS (IBS-C). Probiotic strains varied, with combinations such as *Lactobacillus rhamnosus*, *Bifidobacterium infantis*, and *Saccharomyces boulardii* frequently used. Dosages ranged from 10⁷ to 10¹¹ CFU, with treatment durations spanning two weeks to six months. High-dose multi-strain probiotics were commonly associated with longer interventions (12-16 weeks), reflecting the diversity of therapeutic protocols across the studies (Table 2).

**Table 2:** Types of IBS, diagnostic criteria, probiotic strains, and treatment protocols. IBS: irritable bowel syndrome; NA: not applicable; ATCC: American Type Culture Collection; MTCC: Microbial Type Culture Collection.

Author (Year)	Type of IBS (%)	Diagnostic criteria for IBS	Probiotic	Probiotic dosage	Duration of treatment
Abbas et al. (2014) [13]	Diarrhea (100%)	Rome III	Saccharomyces boulardii	3×10^9^	6 weeks
Jafari et al. (2014) [17]	All types	Rome III	Combination	8×10^9^	4 weeks
Lorenzo et al. (2014) [18]	Diarrhea (100%)	Rome III	Combination	High dose (1-3×10^10^); low dose (3-6×10^9^)	6 weeks
Ludidi et al. (2014) [19]	All types	Rome III	Combination	5×10^9^	6 weeks
Pedersen et al. (2014) [20]	Diarrhea (38%), constipation (17.3%), non-specific (44.7%)	Rome III	Lactobacillus rhamnosus GG	1.2×10^10^	6 weeks
Shavakhi et al. (2014) [21]	Diarrhea (32.6%), constipation (45.7%), non-specific (21.7%)	Rome III	Combination	2×10^8^	2 weeks
Sisson et al. (2014) [22]	Diarrhea (37.6%), constipation (21.5%), non-specific (5.4%), mixed (35.5%)	Rome III	Combination	2×10^8^	12 weeks
Stevenson et al. (2014) [23]	Diarrhea (37.6%), constipation (21.5%), mixed (40.9%)	Rome II	Lactobacillus plantarum 299 v	1×10^10^	8 weeks
Yoon et al. (2014) [24]	Diarrhea (53.1%), constipation (40.8%), mixed (6.1%)	Rome III	Combination	1×10^10^	4 weeks
Faghihi et al. (2015) [25]	Diarrhea (35.3%), constipation (39.6%), mixed (25.1%)	Rome II	Escherichia coli Nissle 1917	NA	6 weeks
de Chambrun et al. (2015) [14]	Diarrhea (28.5%), constipation (46.9%), mixed (24.6%)	Rome III	Saccharomyces cerevisiae CNCM I-3856	4×10^9^	8 weeks
Yoon et al. (2015) [26]	Diarrhea (48.1%), constipation (18.5%), mixed (21.0%), non-specific (12.4%)	Rome III	Combination	1×10^10^	4 weeks
Lyra et al. (2016) [27]	Diarrhea (38.9%), constipation (16.6%), mixed (44.0%), non-specific (0.5%)	Rome III	L. acidophilus NCFM (ATCC 700396)	Low-dose: 1×10^9^; high-dose: 1×10^10^	12 weeks
Majeed et al. (2016) [28]	Diarrhea (100%)	Rome III	Bacillus coagulans MTCC 5856	2×10^9^	90 days
Mezzasalma et al. (2016) [29]	Constipation (100%)	Rome III	1: L. acidophilus, L. reuteri; 2: L. plantarum, L. rhamnosus, B. animalis subsp. Lactis	1: 1×10^10^; 2: 1.5×10^10^	60 days
Spiller et al. (2016) [30]	Diarrhea (20.8%), constipation (47.5%), mixed (31.7%)	Rome III	Saccharomyces cerevisiae I-3856	8×10^9^	12 weeks
Thijssen et al. (2016) [31]	Diarrhea (30%), constipation (25%), mixed (28.75%), non-specific (16.25%)	Rome II	Lactobacillus casei Shirota	1.3×10^10^	8 weeks
Hod et al. (2017) [32]	Diarrhea (100%)	Rome III	Combination	5×10^10^	8 weeks
Ishaque et al. (2018) [33]	Diarrhea (100%)	Rome III	Combination	8×10^9^	16 weeks
Khodadoostan et al. (2018) [15]	Diarrhea (100%)	Rome III	Combination	2×10^9^	6 months
Kim et al. (2018) [16]	NA	NA	Lactobacillus gasseri BNR17	low-dose: 1×10^9^; high-dose: 1×10^10^	4 weeks
Preston et al. (2018) [34]	Diarrhea (46.4%), constipation (35.7%), mixed (18.6%)	Rome III	Combination	1×10^11^	6 weeks
Sun et al. (2018) [35]	Diarrhea (100%)	Rome III	Clostridium butyricum	5.67×10^7^	4 weeks

Symptom relief was measured using validated scales such as Irritable Bowel Syndrome Symptom Severity Score (IBS-SSS), Visual Analog Scale (VAS), and Likert scales. Across the studies, probiotics demonstrated a significant reduction in symptom severity, with reductions in IBS-SSS scores often exceeding 50 points, a clinically meaningful threshold. Improvement rates for abdominal pain and bloating ranged from 30% to 50% in several trials. Global symptom relief, defined as at least a 30% reduction in overall symptom scores, was reported in a majority of studies, highlighting the efficacy of probiotics in IBS management. Adverse events were infrequent and mild, with most participants tolerating probiotics well (Table 3).

**Table 3:** Symptom improvement criteria and outcomes. IBS: irritable bowel syndrome; VAS: Visual Analog Scale; MSS: mean symptom composite score; IBS-SSS: Irritable Bowel Syndrome Symptom Severity Score.

Author (year)	Criteria to determine symptom improvement	Outcome
Abbas et al. (2014) [13]	Continuous scale for IBS symptoms	Abdominal pain (four-point scale), bloating (four-point scale), adverse events
Jafari et al. (2014) [17]	Satisfactory relief of global IBS symptoms for at least 50% of the time	Relief of IBS symptoms, abdominal pain (100-mm VAS)
Lorenzo et al. (2014) [18]	“Considerably relieved” or “completely relieved” of global IBS symptoms for at least 50% of the time	Health-related quality of life (a specific questionnaire ranging from 1 to 100), respond (relief of symptoms)
Ludidi et al. (2014) [19]	A 30% or greater improvement in mean symptom composite score (MSS)	Respond (MSS)
Pedersen et al. (2014) [20]	Continuous scale for IBS symptoms	IBS-SSS
Shavakhi et al. (2014) [21]	Continuous scale for IBS symptoms	Abdominal pain (four-point scale) Distension (four-point scale)
Sisson et al. (2014) [22]	Patients reported mild or no symptoms	Respond (IBS-SSS), abdominal pain (IBS-SSS), bloating (IBS-SSS), adverse events
Stevenson et al. (2014) [23]	Continuous scale for IBS symptoms	IBS-SSS, adverse events
Yoon et al. (2014) [24]	Global relief of IBS symptoms	Global relief of IBS symptoms, abdominal pain (10-point numerical scale), bloating (10-point numerical scale), adverse events
Faghihi et al. (2015) [25]	Continuous scale for IBS symptoms	Global symptoms score (Birmingham IBS Symptom Questionnaire)
de Chambrun et al. (2015) [14]	A reduction in the abdominal pain score of 1 arbitrary unit (au) for at least 50% of the time	Improvement in IBS symptoms, abdominal pain (7-point Likert scale), adverse events
Yoon et al. (2015) [26]	Adequate relief of global IBS symptoms	Adequate relief of global IBS symptoms, global symptoms score (10-point VAS), abdominal pain (10-point VAS) Bloating (10-point VAS)
Lyra et al. (2016) [27]	Continuous scale for IBS symptoms	IBS symptom severity scores (IBS-SSS), abdominal pain (IBS-SSS), bloating (IBS-SSS) Adverse events
Majeed et al. (2016) [28]	Continuous scale for IBS symptoms	Abdominal pain (questionnaire), bloating (questionnaire), adverse events
Mezzasalma et al. (2016) [29]	A decrease in abdominal pain of at least 30% compared to the basal condition for at least 50% of the intervention time	Response (the subject reporting a decrease of symptoms of at least 30% compared to the basal condition for at least 50% of the intervention time)
Spiller et al. (2016) [30]	An improvement of 50% of the weekly average “intestinal pain/discomfort score” compared with the baseline average score for at least 4 out of the last 8 weeks of the study	Response, global symptoms score, abdominal pain (8-point Likert scale), bloating (8-point Likert scale), adverse events
Thijssen et al. (2016) [31]	A mean symptom score (MSS) decrease of at least 30%	Response (a mean symptom score (MSS) decrease of at least 30%)
Hod et al. (2017) [32]	Improvement in symptoms for at least 50%	Response, adverse events
Ishaque et al. (2018) [33]	Continuous scale for IBS symptoms	IBS symptom severity scores (IBS-SSS), abdominal pain (IBS-SSS)
Khodadoostan et al. (2018) [15]	Continuous scale for IBS symptoms	Abdominal pain (10-point VAS)
Kim et al. (2018) [16]	Continuous scale for IBS symptoms	Abdominal pain (5-point Likert scale), bloating (5-point Likert scale)
Preston et al. (2018) [34]	Continuous scale for IBS symptoms	IBS symptom severity scores (IBS-SSS), abdominal pain (IBS-SSS)
Sun et al. (2018) [35]	A reduction of ≥50 points in total IBS-SSS score	Response (a reduction of ≥50 points of total IBS-SSS score), IBS symptom severity scores (IBS-SSS), abdominal pain (IBS-SSS), bloating (IBS-SSS), adverse events

Meta-Analysis

**Figure 4:**
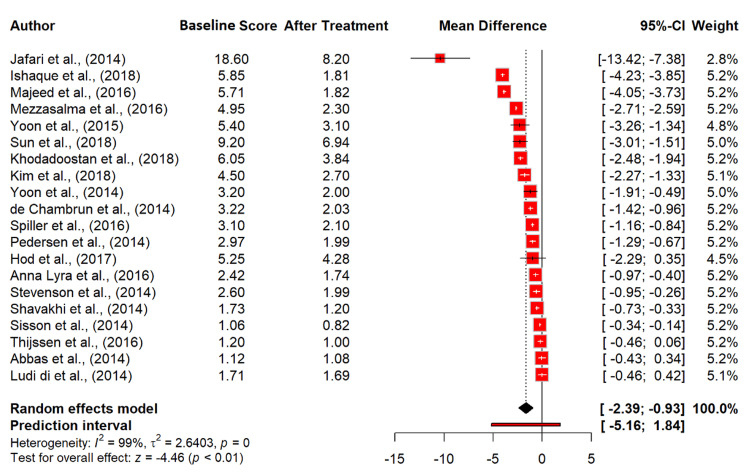
Abdominal pain score in irritable bowel syndrome patients at baseline and after probiotics treatment. References [13-17,19-24,26-33,35].

Our meta-analysis assessed the impact of probiotics on abdominal pain in IBS patients across 20 studies, involving 5,634 observations [13-17,19-24,26-33,35]. The random effects model indicated a significant mean difference (MD) of -1.66 (95% CI: -2.39 to -0.93), with a highly significant p-value of <0.0001, suggesting a substantial reduction in abdominal pain due to probiotic treatment. The heterogeneity was exceptionally high (I² = 99.5%), with a tau² value of 2.6403, indicating significant variability across the studies (Figure 4).

**Figure 5:**
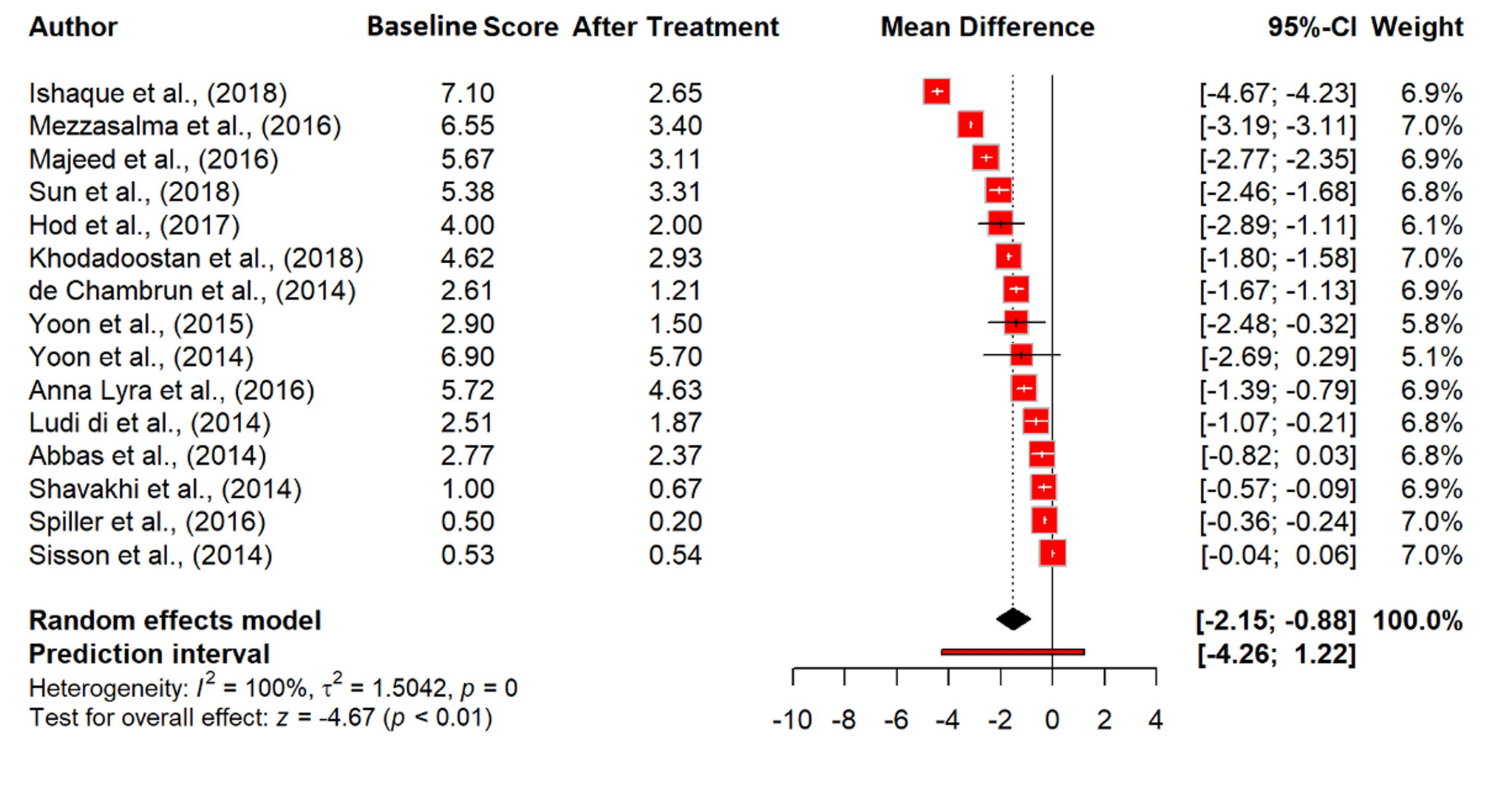
Bowel habits (constipation/diarrhea) score in irritable bowel syndrome patients at baseline and after probiotics treatment. References [13-15,19,21,22,24,26-30,32,33,35].

In 15 studies with 4,850 observations [13-15,19,21,22,24,26-30,32,33,35], probiotics showed a significant improvement in bowel habits, evidenced by a mean difference of -1.52 (95% CI: -2.15 to -0.88) and a p-value of <0.0001. The heterogeneity was nearly complete (I² = 99.9%), highlighting substantial study variability (Figure 5).

**Figure 6:**
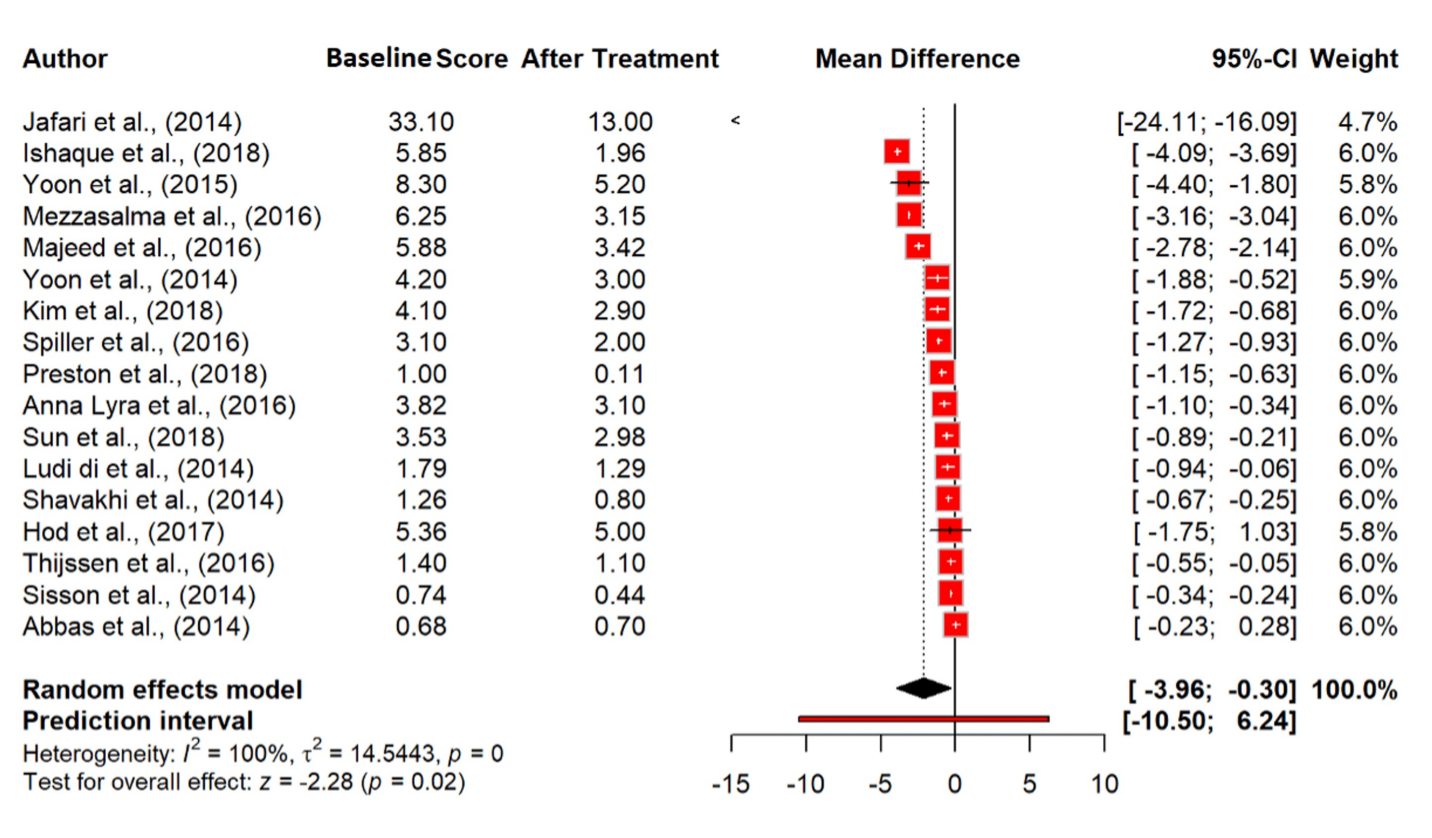
Bloating/abdominal distention/flatus score in irritable bowel syndrome patients at baseline and after probiotics treatment. References [13,16,17,19,21,22,24,26-35].

Analysis of 17 studies [13,16,17,19,21,22,24,26-35] with 5,044 participants revealed that probiotics significantly reduced bloating and abdominal distension with a mean difference of -2.13 (95% CI: -3.96 to -0.30) and a p-value of 0.0224. This analysis also showed extremely high heterogeneity (I² = 99.7%), suggesting diverse effects across different studies (Figure 6).

**Figure 7:**
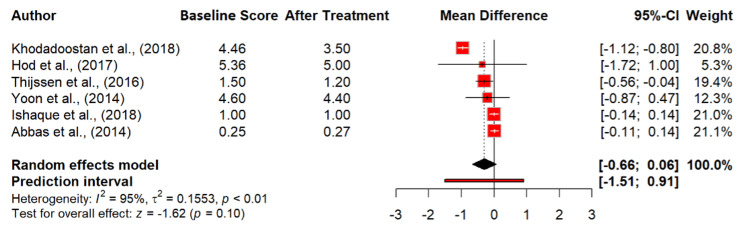
Mucus in stool in irritable bowel syndrome patients at baseline and after probiotics treatment. References [13,15,24,31-33].

Moreover, the effect of probiotics on mucus in stools was analyzed in six studies involving 1,470 participants [13,15,24,31-33]. The result showed a non-significant mean difference of -0.30 (95% CI: -0.66 to 0.06; p = 0.104), indicating that probiotics may not significantly alter mucus production in IBS patients. The heterogeneity remained high (I² = 95.0%), reflecting variability among the studies (Figure 7).

**Figure 8:**
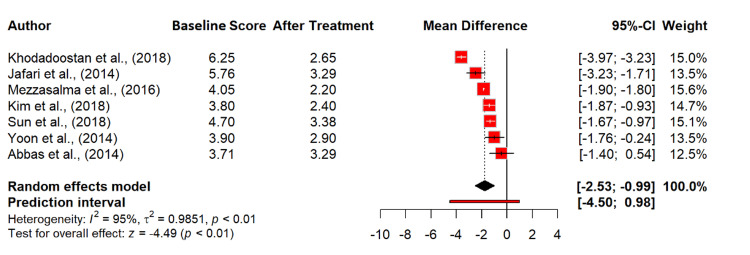
Incomplete evacuation in irritable bowel syndrome patients at baseline and after probiotics treatment. References [13,15-17,24,29,35].

However, seven studies [13,15-17,24,29,35], with 1,376 observations showed that probiotics significantly improved symptoms of incomplete evacuation, with a mean difference of -1.76 (95% CI: -2.53 to -0.99; p < 0.0001). The heterogeneity was high (I² = 94.7%), suggesting variability in the treatment effects across studies (Figure 8).

**Figure 9:**
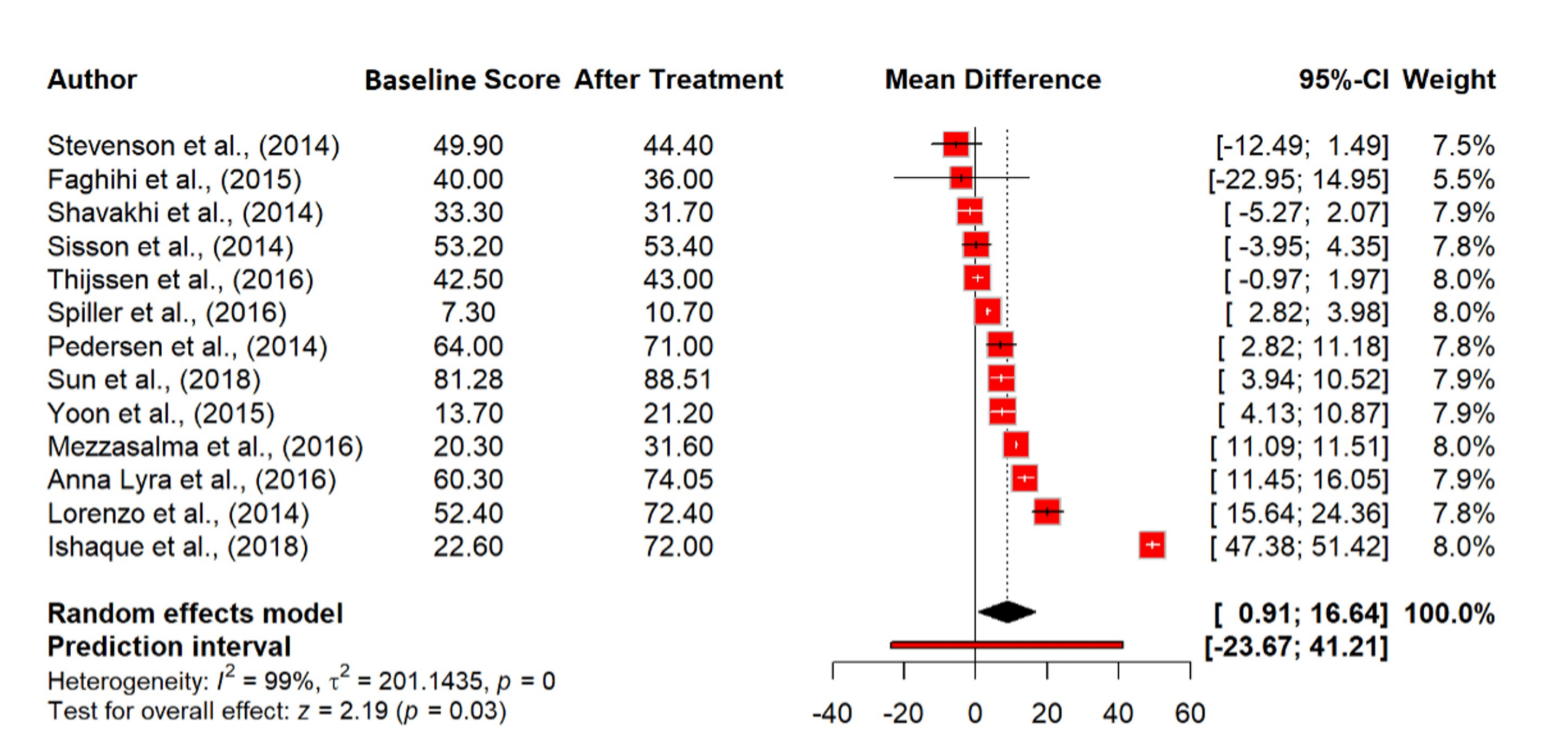
Quality of life (QoL) satisfaction score in irritable bowel syndrome patients at baseline and after probiotics treatment. References [18,20-23,25-27,29-31,33,35].

Finally regarding quality of life (QoL), 13 studies [18,20-23,25-27,29-31,33,35], including 4,680 observations, revealed that probiotics treatment significantly improved QoL satisfaction in IBS patients with a mean difference of 8.77 (95% CI: 0.91 to 16.64; p = 0.028). The extremely high heterogeneity (I² = 99.5%) suggests varied outcomes among different populations or study conditions (Figure 9).

Publication Bias and Heterogeneity

**Figure 10:**
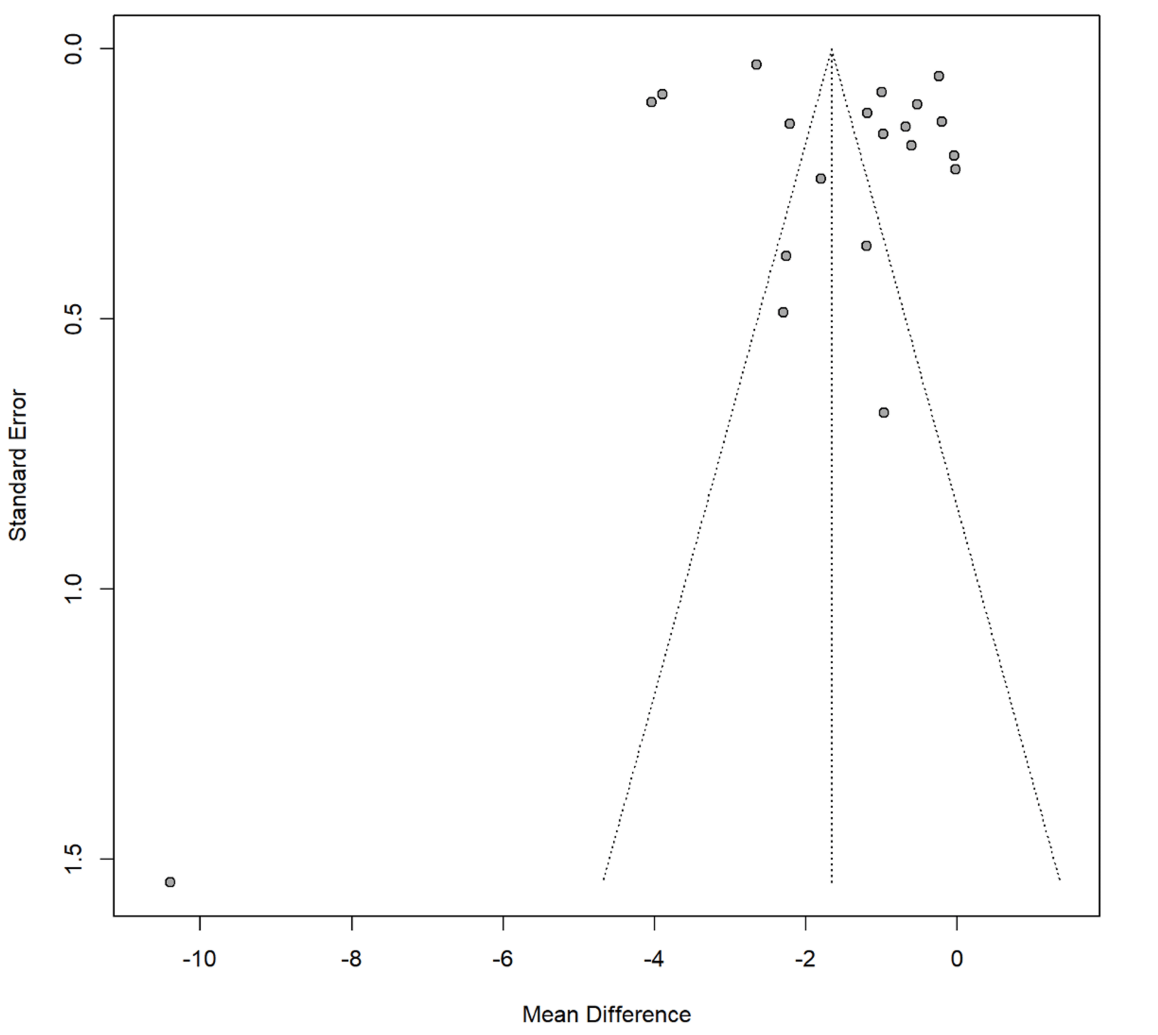
Plot showing publication bias and heterogeneity.

Figure 10 shows potential publication bias, indicated by asymmetry where more studies cluster to the left of the combined mean difference. This suggests smaller studies with significant effects are more likely to be published. High heterogeneity is visually confirmed by the broad spread of studies across the standard error scale, aligning with a reported I² value of 99.5%. This plot highlights the need for careful interpretation of the results, considering possible biases and the variability of study outcomes.

Discussion

IBS is a multifactorial condition significantly impacting the quality of life and probiotics have emerged as a potential therapeutic option due to their effects on gut microbiota modulation. This systematic review and meta-analysis synthesizes evidence from 23 studies to evaluate the efficacy of probiotics in managing IBS symptoms, showing their benefits and limitations.

Probiotics significantly reduce abdominal pain and bloating, with reductions in IBS-SSS scores exceeding clinically meaningful thresholds. These findings were shown by Zhang et al. (2023), who highlighted the efficacy of probiotics, particularly multi-strain combinations, in alleviating IBS symptoms [10]. The observed improvements in global symptom relief in our meta-analysis, noted in most studies, further corroborate the growing consensus on probiotics' role as an adjunct therapy in IBS management [36-38].

One important finding is the variability in treatment efficacy among different IBS subtypes. Diarrhea-predominant IBS (IBS-D) showed the most consistent improvements, particularly with strains like *Saccharomyces boulardii* and *Lactobacillus rhamnosus*. These results resonate with the study by Sun et al. (2018), which also reported pronounced benefits in IBS-D patients using *Clostridium butyricum* [35]. However, the effectiveness of probiotics in constipation-predominant IBS (IBS-C) remains less robust, necessitating further exploration into probiotic regimens for this subgroup [39].

Despite the promising outcomes, the analysis revealed heterogeneity across studies. Differences in diagnostic criteria (e.g., Rome II vs. Rome III), probiotic strains, dosages, and treatment durations contribute to this variability. While high-dose multi-strain probiotics were associated with more significant and sustained improvements, as seen in studies like Lyra et al. (2016) [27], others using single-strain preparations yielded mixed results. This heterogeneity shows the importance of standardizing study protocols and defining optimal probiotic regimens for IBS [40,41].

The safety profile of probiotics was favorable, with infrequent and mild adverse events reported, supporting their use as a generally safe intervention. However, publication bias, indicated by the funnel plot asymmetry, suggests a tendency to report positive results over negative or neutral outcomes. This limitation shows the need for more transparent reporting in future trials.

The impact of probiotics on bowel habits, such as consistency and frequency, was significant but varied among strains. *Lactobacillus plantarum* 299v and *Escherichia coli* Nissle 1917 showed benefits in mixed-type IBS patients, as reported in studies like Faghihi et al. (2015) [25]. These findings align with the studies that reported probiotics exert strain-specific effects, necessitating strain selection based on patient-specific characteristics and IBS subtypes [42,43].

QoL improvements observed in 13 studies were another significant finding. Probiotics enhanced patient satisfaction and symptom management, reflecting their potential to address IBS's psychosocial burden. This aligns with studies emphasizing the benefits of probiotics beyond symptom relief, including reduced stress and anxiety associated with IBS [44,45].

However, the high heterogeneity (I² > 90% in most outcomes) limits the generalizability. Factors such as patient demographics, baseline microbiota composition, and adherence to therapy likely influence treatment outcomes. Future studies should incorporate personalized approaches, combining probiotics with dietary and lifestyle interventions to optimize efficacy.

In comparison with other therapeutic options, probiotics show a non-invasive, cost-effective alternative. Yet, their efficacy in severe cases of IBS or when compared to pharmacological treatments like antispasmodics remains to be conclusively established. The lack of long-term follow-up in most included studies further limits our understanding of probiotics' sustained effects.

This study has several strengths, including its systematic approach to pooling data from a large number of RCTs. By including studies with diverse patient populations and treatment protocols, the findings reflect a wide range of real-world scenarios. However, the study also has limitations. High heterogeneity among studies, caused by variations in probiotic strains, dosages, treatment durations, and IBS subtypes, makes it challenging to generalize the results. Publication bias and the lack of long-term follow-up in most studies may underestimate or overestimate the true efficacy of probiotics.

Future research should focus on standardizing probiotic interventions, including consistent use of diagnostic criteria, well-defined strains, and clear treatment protocols, to reduce variability and improve comparability across studies. Large-scale, long-term trials are essential to assess the sustainability of probiotics' benefits and their effectiveness in different IBS subtypes [11,46]. It is also important to explore personalized approaches, combining probiotics with dietary modifications, like low FODMAP (fermentable oligosaccharides, disaccharides, monosaccharides, and polyols) diets, to optimize outcomes for individual patients. Researchers should prioritize transparent reporting to address publication bias and include diverse populations to enhance the applicability of findings.

## Conclusions

Probiotics are effective in managing symptoms of IBS, particularly in reducing abdominal pain and bloating and improving quality of life. Multi-strain probiotics and higher dosages over longer treatment durations tend to yield better outcomes, especially in diarrhea-predominant IBS. The favorable safety profile of probiotics further supports their use as a non-invasive and accessible treatment option. While probiotics show potential as an adjunct to IBS management, their precise role and optimal use remain unclear. Future research should focus on well-designed, long-term studies with standardized protocols to establish more definitive and personalized treatment strategies.
